# How Important Is Informed Natal Dispersal for Modelling the Demographic and Genetic Effects of Environmental Variability?

**DOI:** 10.1002/ece3.70681

**Published:** 2024-12-16

**Authors:** Sam C. Banks, Ian D. Davies, Teigan Cremona, Hugh F. Davies, Alexander R. Carey, Geoffrey J. Cary

**Affiliations:** ^1^ Research Institute for the Environment and Livelihoods, Faculty of Science and Technology Charles Darwin University Casuarina Northern Territory Australia; ^2^ Fenner School of Environment and Society The Australian National University Canberra Australian Capital Territory Australia

**Keywords:** dispersal, disturbance, genetic diversity, informed dispersal, metapopulation, migration, settlement

## Abstract

Dispersal is a fundamental ecological process that influences population dynamics and genetic diversity and is therefore an important component of the models used to simulate population responses to environmental change. We considered informed dispersal in relation to settlement location, where individuals could optimise selection of settlement location with regard to per capita resource availability and investigated the importance of this type of informed dispersal for simulated demography and genetic diversity under different biological and environmental scenarios. We used an individual‐based simulation model scaled with reference to the ecology of small mammals in fire prone savanna ecosystems. We simulated demographic and genetic processes under informed and uninformed dispersal across several scenarios of life history, environmental heterogeneity (patch size and patch dynamics) and dispersal distance. The effect of the dispersal method on population size far outweighed that of dispersal distance under all combinations of habitat quality, temporal dynamics, patch size and dispersal distance modelled. The effects of habitat patch size and habitat dynamics (representing temporal change in habitat quality such as that potentially generated by disturbance) were low under most of the scenarios modelled. Informed dispersal influenced genetic diversity and differentiation, but the effects were weaker than those of dispersal distance. The genetic effect of informed dispersal occurred through the effect on genetic diversity of the overall metapopulation, while the dispersal distance influenced gene flow and genetic diversity within subpopulations. Informed dispersal was less effective in increasing population size in models of a long‐lived species with overlapping generations. This was particularly true when habitat quality was dynamic and natal dispersal choices did not result in lifelong habitat quality outcomes. Our results suggest that including informed decision‐making in dispersal in simulation models leads to different projections of demography, genetic diversity and susceptibility to environmental change.

## Graphical abstract

Dispersal is a fundamental ecological process that influences population dynamics and genetic diversity and is therefore an important component of the models used to simulate population responses to environmental change. We used a spatially explicit individual‐based model to demonstrate that informed dispersal (in relation to per capita resource availability) can have major effects on abundance and genetic diversity, although this is mediated by temporal habitat dynamics such as those driven by disturbance regimes. Our results suggest that including informed decision‐making in dispersal in simulation models leads to different projections of demography, genetic diversity and susceptibility to environmental change.

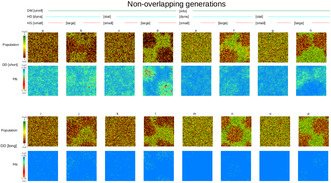

## 1 Introduction

Dispersal has been broadly described as ‘movements potentially leading to gene flow’ (Ronce 2007). A typical scenario is post‐natal dispersal from an individual's birth location to a breeding location (Clobert et al. 2001; Greenwood 1980). It is an individual process that has important consequences for spatial and temporal population dynamics (Clobert et al. 2001) and responses to environmental change (Araújo and Rahbek 2006). For these reasons, dispersal is a crucial component of models used to simulate the responses of populations to environmental processes like disturbance (e.g., fire regimes), pest or feral animal control (Lustig et al. 2019) and, at a global scale, climate change (Brook et al. 2009). Individual‐based models (IBMs) are increasingly used as research tools to help predict the adaptation and persistence of species under different scenarios of environmental change (Xuereb et al. 2021). Hence, understanding the importance of dispersal modelling decisions on simulated patterns of population size and genetic variation is important for the application of IBMs to ecological, genetic and conservation research.

Dispersal is commonly modelled as a simple probability distribution based on distance, called a dispersal kernel (Clark, Macklin, and Wood 1998). However, observed dispersal patterns in natural landscapes vary among individuals and have been documented to be associated with processes relating to the emigration, movement and settlement phases of dispersal events (Bowler and Benton 2005). These factors could be considered the total dispersal kernel (TDK) for animals, analogous to the weighted sum of dispersal vectors for plants (Nathan 2007). Emigration decisions can be associated with condition, sex, genetic diversity, inbreeding risk, resource availability or competition (Stillman et al. 2022). Movement patterns can respond to land cover through impacts on landscape resistance (Spear et al. 2010). Settlement decisions can be influenced by the availability of, and competition for, resources or suitable mates (Clobert et al. 2009). Consequently, the process of dispersal events and pattern of dispersal outcomes are more complex than simple kernel functions (Gilroy and Lockwood 2016; Rogers et al. 2019) and a body of research literature has emerged to focus on how dispersal is represented in spatial population models (Brook et al. 2009; Miller and Holloway 2015).

In this study, we investigate the concept of informed dispersal and whether or not modelling dispersal as an informed process has consequences for simulated patterns of demographic abundance and genetic diversity, with a particular focus on disturbance‐prone environments that drive spatiotemporal changes in habitat quality. Dispersal decisions can be considered ‘informed’ if they are based on any cues such as social or environmental information (Clobert et al. 2009). Our treatment of uninformed versus informed dispersal relates to resource availability, where individuals either select a dispersal destination based on a probability distribution of dispersal distances alone (Uninformed), or select from alternative settlement locations based on distance and per capita resource availability (Informed). Modelling and empirical evidence suggests that the ability to recognise and settle in habitat patches during dispersal can influence metapopulation size and range dynamics (Fronhofer, Nitsche, and Altermatt 2017; Hawkes 2009; Riotte‐Lambert and Laroche 2021; Schmidt and Massol 2019). Our study extends this to evaluate the role of informed dispersal on demography and genetic diversity and under different scenarios of life history and spatiotemporal environmental variation. This presents the opportunity to evaluate how informed dispersal may influence abundance and genetic diversity in heterogeneous landscapes and whether an informed process mediates the effect of dispersal distance on abundance and genetic diversity under those conditions.

We evaluate the outcomes of simulated informed and uninformed dispersal under different scenarios of environmental heterogeneity, including fixed versus temporally dynamic spatial patterns of environmental variation. Spatial and temporal variation in habitat quality is a key driver of dispersal evolution (Duputié and Massol 2013). In turn, dispersal can be important in how animal populations respond to ecological disturbances (Amarasekare and Possingham 2001; Banks et al. 2017). For instance, some ecological and evolutionary simulation models predict that spatial heterogeneity in habitat quality should favour low dispersal, as individuals are typically born in good habitat and emigration leads to a risk of settling in poorer‐quality habitat (Duputié and Massol 2013; Snyder and Chesson 2003). Where spatial heterogeneity in habitat quality changes over time, greater dispersal capability can confer higher survival and increase the likelihood of population persistence (Banks, Davies, and Cary 2017).

We ran a simulation experiment using an individual‐based, spatially explicit model linking spatiotemporal habitat dynamics to demography and genetic diversity to test the effect of dispersal distance and habitat searching behaviour (informed dispersal) on abundance and genetic diversity at the local (grid cell) level and abundance, genetic diversity and genetic differentiation at the landscape level. To understand whether the importance of informed dispersal is moderated by dispersal distance, life history or environmental variability, we compared the effects of informed and uninformed dispersal on these response variables under scenarios of short and long‐distance dispersal, overlapping and non‐overlapping generations, fine‐scale versus coarse‐scale spatial patchiness of habitat quality and a static versus dynamic landscape, where the spatial pattern of habitat quality remained fixed for the entire simulation or varied temporally. The latter scenario is a simple representation of the effects of stochastic spatiotemporal environmental variation on habitat quality. Our simulation model was scaled with reference to the ecological and demographic processes of tropical small mammals in fire‐prone ecosystems from the northern Australian Kapalga fire experiment (Andersen et al. 1998; Griffiths and Brook 2015), and we represented one species with non‐overlapping generations based on the northern quoll ( *Dasyurus hallucatus* ) and one species with overlapping generations based on the northern brushtail possum (*Trichosurus vulpecula arnhemensis*). The model representation of these two species only differ in values for birth and death rates to make them long‐ or short‐lived; both only move in their natal year. This provides the opportunity to test for interactions between time scales of demographic and environmental rates of change.

We hypothesised that:

- Informed dispersal would lead to increased abundance at the landscape level relative to uninformed dispersal due to better settlement choice with respect to resource availability (Dennis, Shreeve, and Van Dyck 2003);
- Greater abundance where mean dispersal distance is high relative to the scale of habitat patchiness (Thomas and Kunin 1999);
- Informed dispersal would have a greater positive effect on abundance where habitat quality is temporally dynamic, but where habitat turnover is not faster than generation times. This is plausible because dispersal takes place only in the natal year and long‐lived species may not benefit as much as short‐lived species when making lifetime decisions in a fast‐changing environment;
- Overall and within‐subpopulation genetic diversity would be higher under informed dispersal due to a lower rate of genetic drift under a larger effective population size;
- Reduced genetic differentiation under informed dispersal relative to uninformed dispersal, even for identical dispersal kernels. We predicted this on the assumption that habitat selection would increase survival of dispersers (and therefore effective dispersal) and possibly lead to increased overall population size; and
- Reduced genetic differentiation under long‐distance dispersal relative to short‐distance dispersal (Bohonak 1999).

Overall, the purpose of this study was to provide insight into how animal movement choices shape population dynamics and genetic diversity and inform the development of models for predicting demographic and genetic patterns under different scenarios of ecological disturbance.

## 2 Methods

We designed simulation experiments using an individual‐based landscape genetics and demographics model (Davies et al. 2016) to test the importance of dispersal distance and habitat searching behaviour (informed dispersal) in models of demography and genetic diversity in heterogeneous landscapes. We evaluated the importance of these aspects of dispersal on population size and genetic diversity under different ecological scenarios relating to the spatial scaling of environmental heterogeneity (large and small patch size) and the predictability of the environment (fixed spatial patterns; and rapid environmental dynamics where patches change every year) (Table 1). We used a generalised linear modelling design (R Core Team 2024) to examine the relative importance of these experimental factors in explaining variation in between‐ and within‐population genetic diversity. We limited our analysis to the relative proportion of variance explained by experimental factors and their interactions, rather than reporting statistical significance (Cary et al. 2006; White et al. 2014).

**TABLE 1:** Experimental design using four factors, each with two levels.

Factor	Symbol	Levels	Details
Dispersal distance	DD	Short; long	100 m; 1000 m. Mean of negative exponential distribution
Dispersal method	DM	Uninformed; informed	Random; best of 3 random trials
Habitat dynamics	HD	Dynamic; static	New pattern every year; constant pattern for entire simulation
Habitat patch size	HS	Small; large	1 ha; 2500 ha

*Note:* Five replicates were performed with simulations lasting 100 years. The total number of simulations was 80.

The model was implemented in the 3Worlds platform (Gignoux, Davies, and Flint 2022) and developed from models used in previous studies of fire, demography and genetic diversity of animal populations (Banks, Davies, and Cary 2017; Davies et al. 2016).

Individuals are located in real (*R*) numbered Cartesian space. Distances for mate searching and dispersal are determined from each individual's location. Competition (for space) is calculated based on an individual's location within a grid (1 ha cells). For the purposes of calculating fixation index, a subpopulation is defined as all individuals found within a square of 3 × 3 cells (9 ha). We have chosen nine cells in order to provide sufficient numbers to estimate allele frequencies for estimation of genetic diversity metrics such as expected heterozygosity.

**FIGURE 1:**
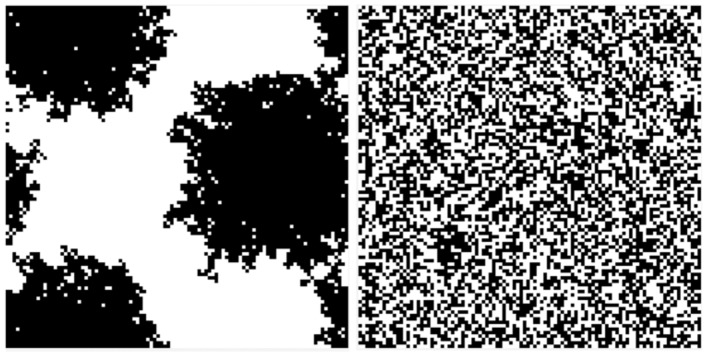
Large and small habitat patches of high and low carrying capacity (white: 3 ha^−1^; black: 1.5 ha^−1^). The entire landscape is 10,000 ha; large patches are approximately 2500 ha and small 1 ha. Small patches are randomly located, often resulting in contiguous patches larger than 1 ha.

The execution order of methods in the simulation loop was as follows:

- *Generate habitat layer*: This occurred every year for scenarios with a dynamic landscape or just once for scenarios where the landscape pattern remained unchanged over the simulation (static). The algorithm divided the landscape into 10,000 cells of 100 × 100 m each with either high or low habitat quality, manifested as variation in carrying capacity (3 and 1.5 individuals per hectare, respectively). The shape and size of habitat patterns were generated to produce irregular circular shapes of approximately 2500 ha (large patch size scenario) or randomly placed 1 ha cells (small patch size scenario) (Figure 1).

ii *Reproduction*: For both short‐ and long‐lived species, mating pairs were randomly selected within a 200 m radius around each female. This distance approximates the scale of the average home range in the less‐dispersive of our reference species ( *Trichosurus vulpecula* ) (Kerle 1998) and the scale of mate choice in congeneric species (Blyton et al. 2016). We made no assumptions about mating fidelity. Offspring were then created and initialised with randomly selected alleles from each parent. No reproduction occurred in the first year of simulation as all individuals are newborns. The birth rate for species with overlapping generations was 0.8 per annum, while that of species with non‐overlapping generations was 2.5 per annum. These values are slightly lower than the corresponding annual estimates of recruitment to the breeding population for the northern brushtail possum and northern quoll, respectively (Griffiths and Brook 2015). However, we used the minimum values required to ensure the populations remain extant over all study scenarios and replicates. We took this approach to provide maximum sensitivity to the experiment factors.
iii *Mortality*: Mortality was then applied to non‐newborn individuals: 100% for non‐overlapping generations and 22% per annum for scenarios with overlapping generations, with a maximum age of 12 years. These scenarios broadly represented the life histories of the semelparous northern quoll and iteroparous northern brushtail possum, respectively (Griffiths and Brook 2015).
iv *Dispersal*: We set dispersal to occur only in the natal year, reflecting common natal dispersal scenarios in mammals. A site for dispersal was selected from a negative exponential distribution (*d* = −*m* log(1—*e*)). The mean distance was either 100 m (short) or 1000 m (long), corresponding to one and 10 cells, respectively, with a random direction. The low end of this range is constrained by the resolution of the habitat layer (1 ha), as many dispersers are removed due to over‐crowding in the natal grid cell. The upper limit approximates the maximum observed movement distances for the northern quoll (Begg 1981). However, sensitivity analysis showed little difference for mean dispersal distances beyond 1000 m. For treatments employing informed dispersal, three dispersal sites were randomly selected from this distribution and the site with the lowest population size relative to carrying capacity was chosen. We limited the number of choices to three based on exploratory simulations (little difference was observed with 10 choices so we chose the computationally efficient option) and research suggesting that a small number of ‘prospecting’ events is favoured in evolutionary models of informed dispersal (Ponchon et al. 2021). To remove edge effects, dispersal took place in a topologically infinite landscape (i.e., a torus) (Haefner et al. 1991).
v *Limit to carrying capacity*: Individuals were then removed from the simulation with a probability proportional to the excess of the population over carrying capacity in each cell. In the case of overlapping generations, new recruits are no more likely to die from crowding than older individuals. Note, it was only through limiting to carrying capacity (without an age‐class bias) that habitat quality effected demography. That is, there was no habitat quality effect on rates of reproduction or mortality at other life stages.
vi *Output individual data*: Measures of population abundance and genetic diversity were calculated including expected and observed heterozygosity overall and within local subpopulations and *F*_ST_ overall and among subpopulations (individuals aggregated over 3 × 3 cells to ensure a sufficiently large sample size for analysis). The method of calculating *F*_ST_ is that found in Davies et al. (2016).
vii *Age*: The model uses yearly time‐steps and age increments by one at the end of the loop.

The model was initialised with 8000 newborn (age 0) individuals (50% female) placed at random locations. Each diploid individual was randomly assigned 1 of 10 alleles at 5 loci that followed a neutral *k*‐allele model.

**FIGURE 2:**
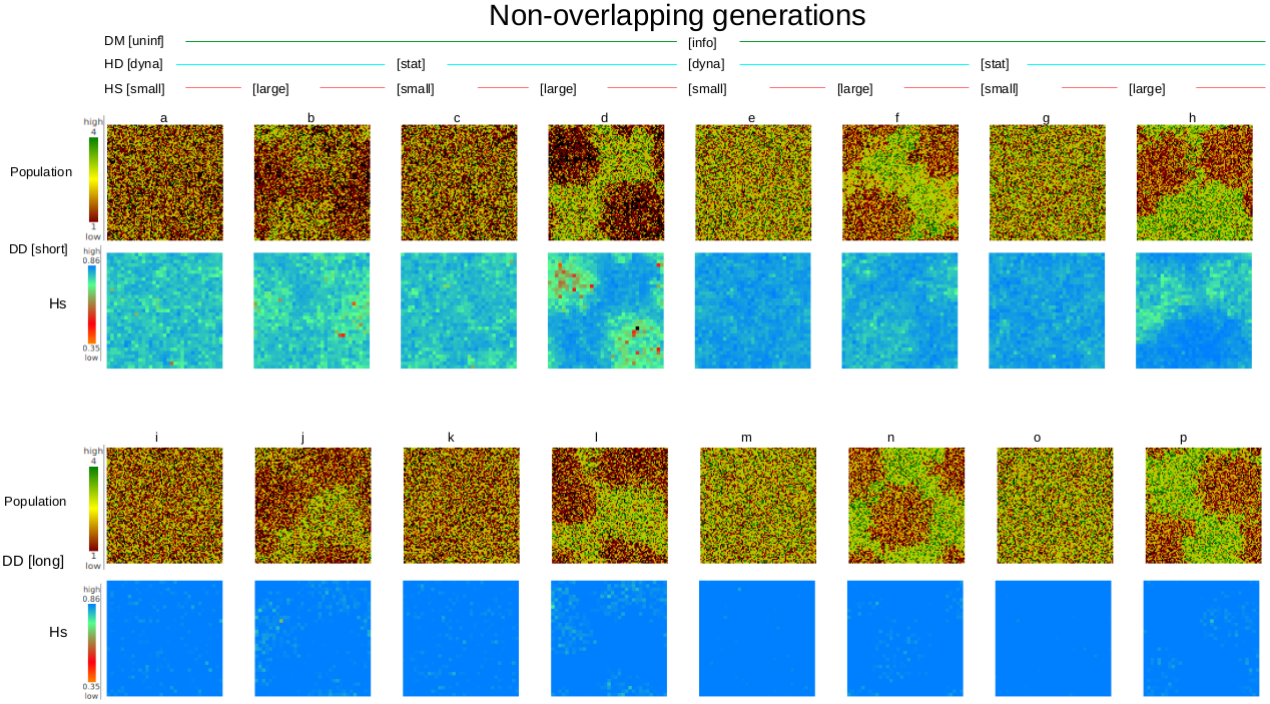
Simulation landscape after 100 years for a population with non‐overlapping generations with 16 scenarios (a–p). Rows are in pairs of (a) population size (1 ha resolution) and (b) heterozygosity (9 ha resolution). The top two rows (pairs a–h) display scenarios using short‐distance dispersal and the bottom two rows (i–p) long‐distance dispersal.

To illustrate the spatial structure of the demographic and genetic components of the model, we have presented single‐generation ‘maps’ of local population size and expected heterozygosity for each simulation scenario in Figures 2 (non‐overlapping generations) and 3 (overlapping generations).

**FIGURE 3:**
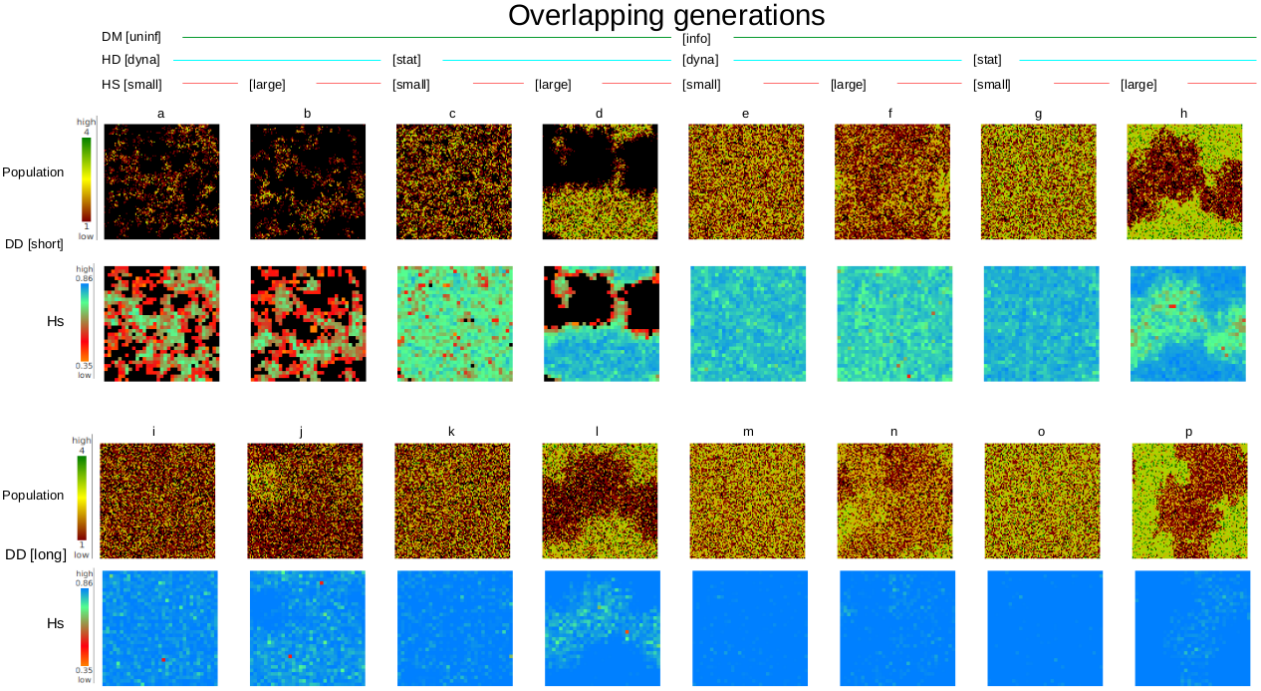
Simulation landscape after 100 years for a population with overlapping generations with 16 scenarios (a–p). Rows are in pairs of (a) population size (1 ha resolution) and (b) heterozygosity (9 ha resolution). The top 2 rows (pairs a–h) display scenarios using short‐distance dispersal and the bottom 2 rows (i–p) long‐distance dispersal.

## 3 Results

### 3.1 Generation Overlap Comparison

We pooled results from simulations with either overlapping or non‐overlapping generations to evaluate the effects of generational overlap on metapopulation size, genetic diversity and structure relative to the other treatments. The choice of overlapping and non‐overlapping generations explained 3% of the variation in both metapopulation size (Figure S1) and *F*_ST_ (Figure S2). Thus, generational overlap was relatively unimportant compared to the other experimental treatments (below), although differences in patterns can be seen in Figures 2 and 3, especially for scenarios (f) and (n), where natal dispersal of long‐lived species does not track habitat quality as well as short‐lived species. Nevertheless, we present the results from the two types of simulations (overlapping and non‐overlapping) separately as there was some nuance in the effects of dispersal distance, dispersal method and habitat dynamics on simulated metapopulation size and genetic diversity patterns among the two experiments.

### 3.2 Non‐Overlapping Generations

**FIGURE 4:**
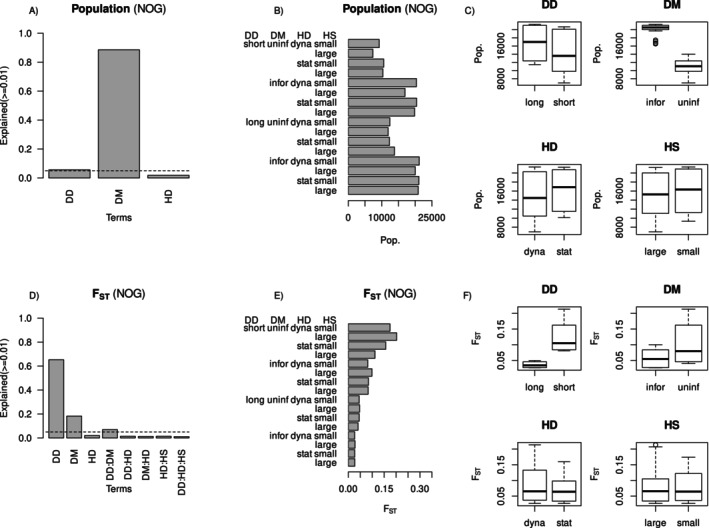
Relative variance explained in (A) population size and (D) *F*_ST_ by dispersal distance (DD), dispersal method (DM) and habitat dynamics (HD) for non‐overlapping generations (NOG). Dotted lines mark 5% of variance explained. Mean population size and *F*_ST_ values are shown across treatments for non‐overlapping generations scenarios in panels (B, E), respectively. Panels (C, F) contrast population size and *F*_ST_ values across the major treatment comparisons including dispersal distance (DD), dispersal method (DM), habitat dynamics (HD) and habitat patch size (HS).

Dispersal method and dispersal distance explained 89% and 6% of the variance in metapopulation size, respectively (Figure 4). Informed dispersal led to almost doubling the population compared to otherwise equivalent scenarios (Figure 4). Metapopulation sizes were generally larger under long‐distance dispersal relative to short‐distance dispersal (Figure 4), but the effect was much weaker than the effect of the dispersal method. Habitat dynamics showed little explanatory power, and habitat patch size was not significant. Habitat patch size showed a significant, though relatively unimportant role in explaining variance in population size (< 1%) (not shown) with small patch size favouring greater abundance for most scenarios (Figure 4).

The ranking of dispersal method and dispersal distance was reversed for explaining variance in *F*_ST_, with dispersal distance and dispersal method explaining 65% and 18% of variance in *F*_ST_, respectively (Figure 4). Here, both short‐distance dispersal and uninformed dispersal produced a metapopulation with greater genetic differentiation among subpopulations (Figure 4).

**FIGURE 5:**
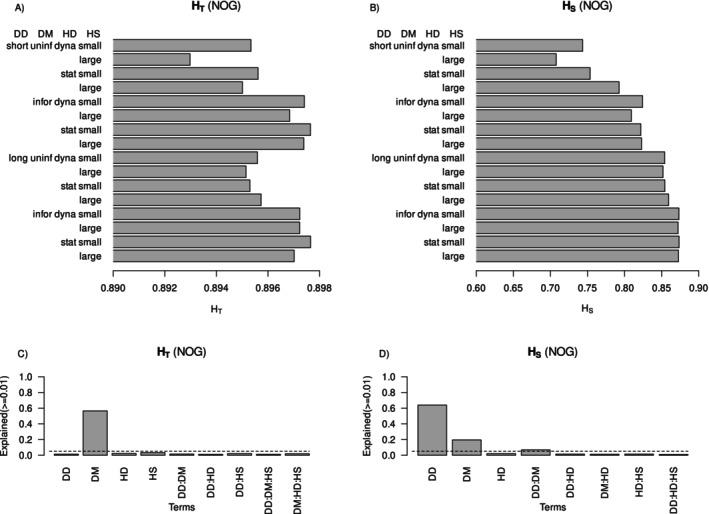
Mean values of expected heterozygosity across the entire simulated metapopulation (*H*_T_) and mean subpopulation expected heterozygosity (*H*_S_) for simulated species with non‐overlapping generations. Panels (A, B) show mean values, and panels (C, D) show variance explained in the two response variables from the major simulation experiment treatments. Dotted lines mark 5% of variance explained.

This greater genetic differentiation was mostly due to reduced mean heterozygosity within subpopulations (mean *H*_S_: Figure 5), noting that overall heterozygosity across the simulation landscape (*H*_T_) was also slightly reduced but to a minor extent under short‐distance or uninformed dispersal (Figure 5). Dispersal method explained most of the variance in *H*_T_, but dispersal distance was the most important experimental treatment influencing mean *H*_S_ (Figure 5).

In the informed dispersal scenarios, the realised dispersal distances tended to be slightly greater (approximately 10% greater) than the proposed mean when dispersal distance was short, especially in a dynamic landscape with a small patch size. This suggests that the ‘best’ place to settle, out of the three simulated choices for each individual, was typically the furthest away when dispersal capability is low and indicates a minor confounding of the dispersal distance and dispersal method treatments under these conditions. We present these results graphically in Figures S1A–S3A with other parameters that enabled interpretation of demographic processes, including realised versus expected dispersal distance, proportion of surviving settlers and proportion of mature females that fall pregnant in each year.

### 3.3 Overlapping Generations

**FIGURE 6:**
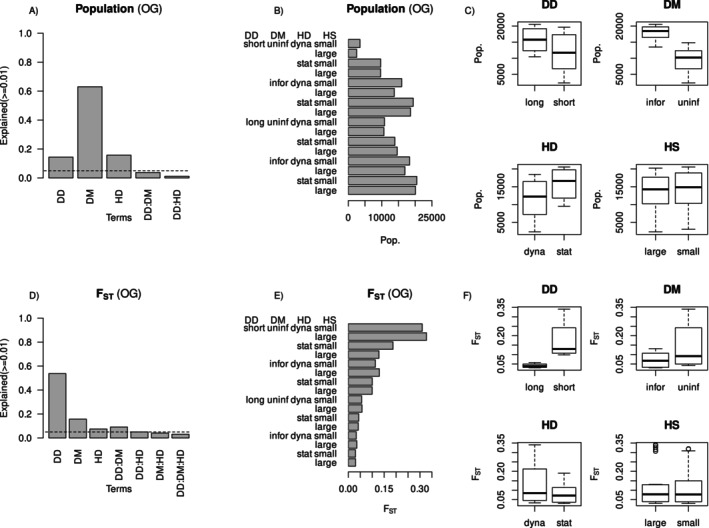
Relative variance explained in (A) population size and (D) *F*_ST_ by dispersal distance (DD), dispersal method (DM) and habitat dynamics (HD) for overlapping generations (OG). Dotted lines mark 5% of variance explained. Mean population size and *F*_ST_ values are shown across treatments for overlapping generations scenarios in panels (B, E), respectively. Panels (C, F) contrast population size and *F*_ST_ values across the major treatment comparisons including dispersal distance (DD), dispersal method (DM), habitat dynamics (HD) and habitat patch size (HS).

Dispersal method and dispersal distance explained 63% and 14% of variance in metapopulation size, respectively (Figure 3), in the same rank order as the experiment with populations with non‐overlapping generations (Figure 4). However, habitat dynamics was also an important explanatory factor (16%) for simulations with longer lifespan and overlapping generations, with a static landscape leading to a larger metapopulation size than a dynamic landscape (Figure 6).

Again, as for populations with non‐overlapping generations, habitat patch size showed a significant, though relatively unimportant, role in explaining variance in population size (< 1%) (not shown) with small patch size favouring greater abundance for the majority of scenarios (Figure 6).

The metapopulation became very small (and would likely have become extinct for longer simulation runs) when dispersal was short and uninformed, and habitat was dynamic, regardless of patch size (Figure 6). Thus, the population became stable if either dispersal was informed, long distance, or the habitat was static (Figure 6).

**FIGURE 7:**
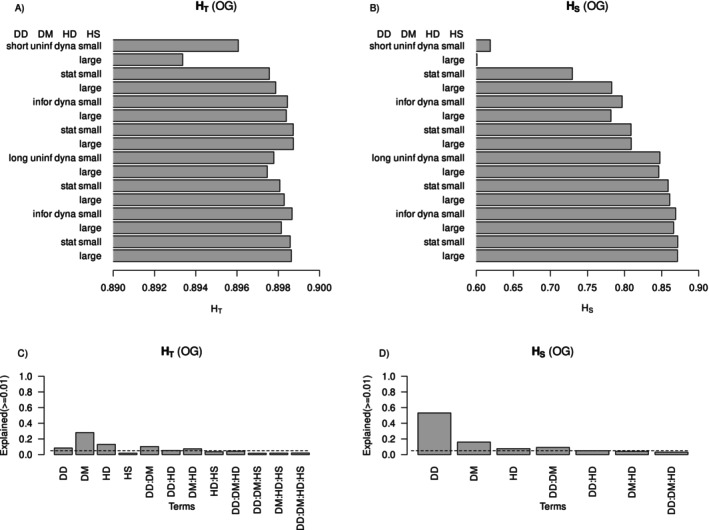
Mean values of expected heterozygosity across the entire simulated metapopulation (*H*_T_) and mean subpopulation expected heterozygosity (*H*_S_) for simulated species with non‐overlapping generations. Panels (A, C) show mean values, and panels (B, D) show variance explained in the two response variables from the major simulation experiment treatments. Dotted lines mark 5% of variance explained.

Variance in *F*_ST_ explained by dispersal distance, dispersal method and habitat dynamics was 54%, 16% and 7%, respectively (Figure 6). Investigating the components of *F*_ST_—expected heterozygosity over the entire metapopulation and the mean expected heterozygosity within subpopulations—dispersal method explained most of the variance in *H*_T_, but dispersal distance was the most important experimental treatment influencing mean *H*_S_ (Figure 7). A dynamic landscape produced a metapopulation with greater genetic differentiation among subpopulations than a static landscape (Figure 3). The major patterns of variation in *F*_ST_ among treatments were associated with a much lower mean *H*_S_ (expected heterozygosity within subpopulations) in scenarios with uninformed dispersal, low dispersal distance and dynamic habitat (Figure 7).

Consistent with the non‐overlapping generation simulations, the realised dispersal distances tended to be slightly greater (approximately 5%–10% greater) than the proposed mean when dispersal distance was short, especially in a dynamic landscape with a large patch size. We present these results graphically in Figures S1B–S3B with other parameters that enabled interpretation of demographic processes, including realised versus expected dispersal distance, proportion of surviving settlers and proportion of mature females that fall pregnant in each year.

## 4 Discussion

Using a dispersal method focused on reducing competition for space inevitably led to a larger metapopulation with greater genetic variation within subpopulations and correspondingly lower genetic differentiation among subpopulations. Thus, failure to take account of an animal's ability to make ‘sensible’ settlement decisions with regard to resources would likely lead to under‐estimation of the population's viability. Under the conditions simulated, dispersal method (informed dispersal) had a far greater effect than dispersal distance on metapopulation size and a weaker effect than dispersal distance on the genetic structure and moderated the effects of simulated environmental heterogeneity on population size and genetic diversity. The effects of informed dispersal on these outcomes differed slightly for short‐lived species with non‐overlapping generations compared to longer‐lived species with overlapping generations, noting that the lifespan of the non‐overlapping generation species simulated was equivalent to the rate of habitat turnover.

### 4.1 Metapopulation Size

A number of modelling studies have predicted that dispersal distance is important for metapopulation size and persistence in spatially or spatiotemporally varying environments and that environmental variability is an important driver of selection on dispersal traits (Amarasekare and Possingham 2001; Duputié and Massol 2013; Snyder and Chesson 2003). Our models provided some support for long‐distance dispersal, leading to greater metapopulation size, especially under dynamic habitat scenarios, but the effect of dispersal method far outweighed that of dispersal distance under all of the combinations of habitat quality, temporal dynamics, patch size and dispersal distance modelled.

We did not identify strong interactions between dispersal distance and either patch size or habitat dynamics (temporal heterogeneity), in relation to outcomes for metapopulation size. Models and microbial experiments show that an intermediate dispersal distance is important for population spread into an unoccupied habitat due to the trade‐off between colonisation distance and the probability of failing to establish at range margins due to Allee effects (Pires and Queirós 2019; Smith et al. 2014). Research on other species suggest that dispersal and colonisation are different behaviours (Simmons, Thomas, and Olivieri 2004) and the Allee effects that counteract the benefits of long‐distance dispersal during colonisation may not occur under other scenarios. In our simulated system, the population was in a dynamic equilibrium, but we acknowledge that there may be combinations of dispersal distance, patch size and patch turnover rate that might yield different outcomes. For instance, Riotte‐Lambert and Laroche (2021) simulated dispersal movements via random walk or straight‐line movement through landscapes and found that a hump‐shaped dispersal distance pattern maximised metapopulation size in the absence of the ability to detect and settle in a suitable habitat but that metapopulation size increased monotonically with dispersal capacity when individuals could detect a suitable habitat.

### 4.2 Genetic Variation

The effects of dispersal distance and gene flow on genetic variation within and among populations are well‐established in population genetics theory and empirical observation (Bohonak 1999), yet this study demonstrates that other components of dispersal behaviour (informed dispersal) can influence spatial patterns of genetic variation through their effects on genetic drift. In our models, the overwhelming factor determining genetic variation within subpopulations (expected heterozygosity) is dispersal distance. These findings stand regardless of generation‐overlap times (1–12‐year lifespan), the extreme range of landscape dynamics (50% change every year to no change at all) and the large range in patch size used in our simulations. However, we found that, for a given mean dispersal distance, informed dispersal was associated with increased genetic diversity within subpopulations and over the entire metapopulation. This effect was weaker than that of dispersal distance but stronger than any of the other treatments in the simulation experiment.

The effect of informed dispersal on modelled patterns of genetic variation invokes a simple biological explanation (consistent with the effects on abundance) and a methodological caveat. Larger mean subpopulation and metapopulation size is expected to reduce the rate of genetic drift (Kimura and Ohta 1969), and we found the informed dispersal scenarios to have larger population sizes at both levels, as well as greater mean expected heterozygosity within subpopulations and (to a lesser extent) over the entire metapopulation, corresponding to a lower level of genetic differentiation (*F*_ST_) among subpopulations. However, we acknowledge a minor confounding of dispersal method and dispersal distance that was particularly apparent under scenarios of short distance dispersal, where informed dispersal on average led to a choice of settlement location slightly further from the natal origin than when settlement was uninformed in relation to resource availability.

Under some circumstances, the distinction between informed and uninformed dispersal led to different predictions about the effects of environmental variability on genetic diversity (and population size). This was particularly apparent where dispersal distance was short and dispersal was uninformed, when both population size and genetic diversity were greatly reduced in dynamic habitat relative to static spatial heterogeneity in habitat quality. Our interpretation of this phenomenon is that the combination of colonisation ability and demographic rates in these models results in a ‘habitat tracking’ scenario that is barely adequate to maintaining metapopulation viability in dynamic habitat. Relatively extreme genetic effects (low heterozygosity within subpopulations and high *F*_ST_) are therefore due to a population dynamic characterised by regular founder events. However, informed dispersal with regard to carrying capacity provides far greater demographic and genetic resilience and precludes the regular ‘small founder event’ scenario. A range of scenarios of habitat and population turnover and founder events driven by environmental change have been documented in natural ecosystems, and differing levels of genetic diversity and differentiation have been observed in association with these dynamics. Those scenarios characterised by low colonisation rates and strong habitat limitation are typically associated with a greater genetic structure and reduced genetic variation within populations (Brown et al. 2013; Derycke et al. 2007; Haileselasie et al. 2018).

### 4.3 Life History Contrasts

As we have set dispersal to take place only in the natal year, long‐lived individuals spent all but their first year in an unpredictable environment. This is likely why the effect of habitat dynamics is more marked for populations with overlapping generations (Figures 3 and 6). In general, populations with non‐overlapping generations are more robust in these experiments (Figures 2 and 3) because resource‐based dispersal decisions are more accurate within the scale of their lifespan. Simulated populations of species with overlapping generations were only marginally viable in a dynamic habitat without either informed dispersal or long‐distance dispersal, although strategies that mitigate the severity of environmental change on demography, or the ability to migrate to track ongoing changes in habitat suitability occur and would benefit such species. Some examples can be drawn from studies of contrasting mammal responses to severe fire in southeastern Australian forests. Fire events have severe effects on the abundance of the short‐lived bush rat ( *Rattus fuscipes* ), yet it recovers rapidly via local recolonisation and ground‐level vegetation post‐fire regeneration (Banks et al. 2017). The mountain brushtail possum ( *Trichosurus cunninghami* ) has overlapping generations and commonly lives for 8–10 years, and the effects of the same fire event that severely affected the bush rat were relatively minor (Banks et al. 2015). The mountain brushtail possum has weaker dispersal capability but has behavioural traits that mitigate the severity of fire events on demography. Like our simulated species, these two species have a strategy of natal dispersal but do not appear to use any other resource‐based migration strategies. Incorporating such behaviours into our models would likely have changed the simulated effect of dynamic habitat quality on long‐lived species with overlapping generations.

### 4.4 Conclusions and Future Directions

A logical question is whether individual‐based simulation models of demographic or genetic processes in natural populations should incorporate informed dispersal. Decisions about model complexity need to be made on a case‐by‐case basis but, given that the dispersal method was the most influential variable in our simulation experiment, the implications of modelling dispersal as informed or uninformed are important and need to be considered.

We focused on informed dispersal that responds to per capita resource availability, but dispersal decisions based on different cues may be equally or more relevant in models that focus on questions other than environmental heterogeneity. For instance, inbreeding avoidance, mate availability or other aspects of conspecific attraction have been documented as influencing dispersal decisions (Banks and Lindenmayer 2014; Gilroy and Lockwood 2016; Hawkes 2009). An important consideration in deciding the level of model complexity is whether omitting informed dispersal either leads to underestimation of size and genetic variation or requires compensation by inflating other model parameters to maintain population viability for simulation studies. Our models featured a simple scenario of two levels of habitat quality that each cover 50% of the landscape. These patterns can change randomly each year or remain fixed for the entire simulation. It may be the case that informed dispersal is less influential in less extreme scenarios of environmental heterogeneity.

## Author Contributions

**Sam C. Banks:** conceptualization (equal), project administration (equal), supervision (equal), writing – original draft (equal). **Ian D. Davies:** conceptualization (equal), formal analysis (lead), software (lead), writing – original draft (equal). **Hugh F. Davies:** conceptualization (equal), writing – review and editing (equal). **Teigan Cremona:** conceptualization (equal), writing – review and editing (equal). **Alexander R. Carey:** conceptualization (equal), writing – review and editing (equal). **Geoffrey J. Cary:** conceptualization (equal), writing – original draft (equal).

## Conflicts of Interest

The authors declare no conflicts of interest.

## Statement on Inclusion

The broader project within which this simulation study was conducted has a major empirical component, with research being conducted in partnership with local stakeholders in government and First Nations organisations and communities in northern Australia. The authorship of this manuscript is limited to a subset of the academic researchers on the project, as it describes research on the technical representation of biological processes in simulation models, which is several steps removed from direct relevance and meaningful engagement.

## Supporting information


Data S1.

## Data Availability

All code and data to reproduce the results of the study have been submitted along with the manuscript (files named ‘model.zip’, ‘outputs.zip’ and ‘readme.odt’) and will be made publicly available through a digital repository prior to manuscript publication.
