# Supplementary material for: How Important Is Informed Natal Dispersal for Modelling the Demographic and Genetic Effects of Environmental Variability?
Source: Ecol Evol. 2024 Dec 16;14(12):e70681. doi: 10.1002/ece3.70681 (PMC11650744; doi:10.1002/ece3.70681)
Supplement: Supplementary file 1 — Data S1. [file ECE3-14-e70681-s001.zip › BCD2023_Online_Material.docx]

Online supplementary material for “How important is informed natal dispersal for modelling the demographic and genetic effects of environmental variability?”

Figure S1. Relative difference in realised dispersal and mean of the dispersal kernel used for simulations in models using non-overlapping and overlapping generations. Under scenarios of short distance dispersal, the ability to select destination based on per capita resource availability led to a slight increase in dispersal distance.

| 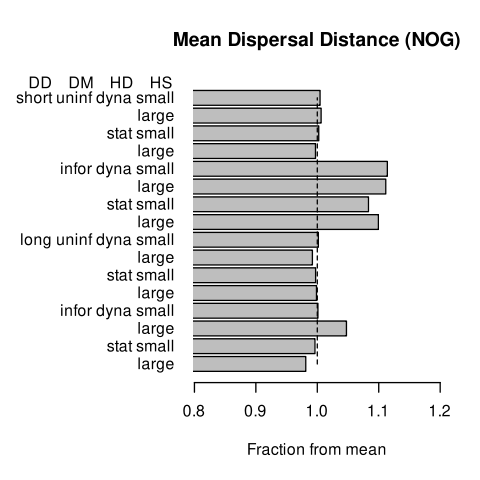 | 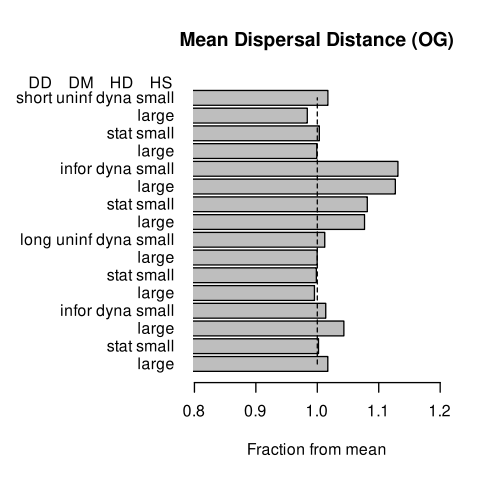 |
| --- | --- |
| Figure S1A. Relative difference in dispersal distance to the dispersal kernel mean for populations with non-overlapping generations. | Figure S1B. Relative difference in dispersal distance to the dispersal kernel mean for populations with overlapping generations. |

Figure S2. The proportion of first-year individuals that survive in each generation. This was used as an indicator for pre-breeding settlement success.

| 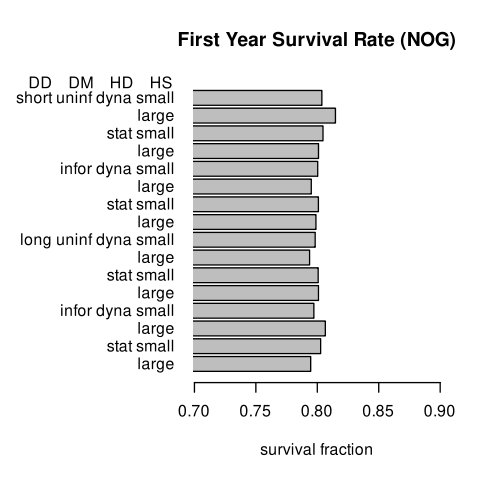 | 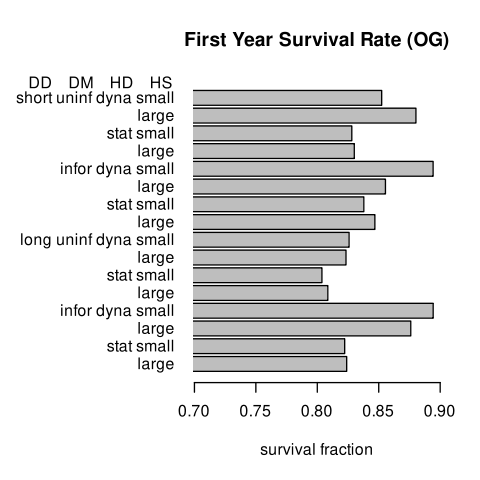 |
| --- | --- |
| Figure S2A. First year survival rate for populations with non-overlapping generations. This is a surrogate for the proportion of individuals that ‘effectively’ disperse. | Figure S2B. First year survival rate for populations with overlapping generations. This is a surrogate for the proportion of individuals that ‘effectively’ disperse. |

Figure S3. The proportion of mature females in non-overlapping (A) and overlapping (B) generations scenarios that fall pregnant under each parameter combination.

| 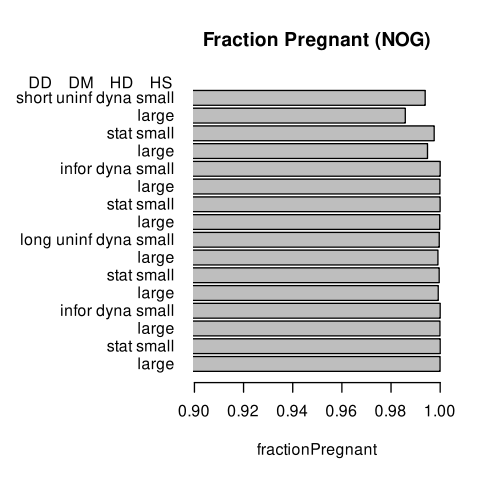 | 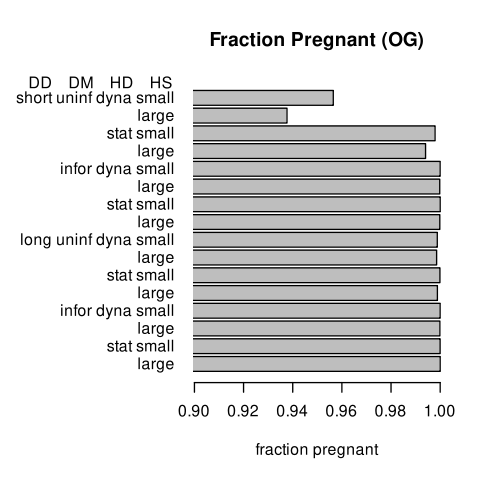 |
| --- | --- |
| Figure S3A. The proportion of mature females that fall pregnant for each parameter combination for populations with non-overlapping populations. | Figure S3B. The proportion of mature females that fall pregnant for each parameter combination for populations with overlapping populations. |
