# Supplementary material for: How Important Is Informed Natal Dispersal for Modelling the Demographic and Genetic Effects of Environmental Variability?
Source: Ecol Evol. 2024 Dec 16;14(12):e70681. doi: 10.1002/ece3.70681 (PMC11650744; doi:10.1002/ece3.70681)
Supplement: Supplementary file 3 [file ECE3-14-e70681-s001.zip › Readme.odt]

Supplementary data

Files

outputs.zip: contains the output data and R scripts used to generate charts for this paper.

model.zip: Dispersal model

To re-run the experiment (a computer with at least 8 cores is recommended):

- Install Java Development Kit (JDK) version 11 at [https://openjdk.org/projects/jdk/11/](https://openjdk.org/projects/jdk/11/). This may require administrator privileges if you are working on a managed workplace computer;

- Create a directory called ‘3w’ in your home directory;

- The version of 3Worlds required to run this experiment is 1.0.3. At the time of submission, it can be downloaded at [https://dropsu.sorbonne-universite.fr/s/T38YDt4wTPHYfTW](https://dropsu.sorbonne-universite.fr/s/T38YDt4wTPHYfTW). 
Later, the software will be available at  [https://zenodo.org/record/7038145](https://zenodo.org/record/7038145). Download the jar suited to your operating system (Linux, Mac or Win);

- Place the software in the root of ‘3w’;

- Unzip model.zip;

- To open and run the experiment, open a terminal and navigate to ‘3w/’;

- Execute the command: java -jar tw<your os>.jar: where ‘<your os>’ is either linux, mac or win. This will start the ModelMaker application;

- When the ModelMaker starts, from the Projects menu, select Import and import DispersalPaper.utg. You will then be prompted to also import Utilities3A.java. Both of these files are in model.zip. A new sub-directory within ‘3w’ will appear called ‘project_DispersalPaper1…’. This will contain output data when the experiment completes;

- To run the experiment, click ‘Deploy’;

- The ModelRunner application will start and initialise 80 simulators. When this finishes, click the forward arrow button (‘>’) to execute the experiment.
